# Lessons Learned: It Takes a Village to Understand Inter-Sectoral Care Using Administrative Data across Jurisdictions

**DOI:** 10.23889/ijpds.v3i3.440

**Published:** 2018-11-12

**Authors:** Patti Ann Groome, Mary L McBride, Li Jiang, Cynthia Kendell, Kathleen M Decker, Eva Grunfeld, Monika K Krzyzanowska, Marcy Winget

**Affiliations:** 1 Division of Cancer Care and Epidemiology, Cancer Research Institute, Queen’s University, Kingston, Ontario Canada; 2 Cancer Control Research, BC Cancer Agency, Vancouver, British Columbia, Canada; 3 Critical Care Services Ontario, Toronto, Ontario, Canada; 4 Cancer Outcomes Research Program, Dalhousie University and Nova Scotia Health Authority, Halifax, Nova Scotia, Canada; 5 Department of Community Health Sciences, University of Manitoba, Winnipeg, Manitoba, Canada; 6 Epidemiology and Cancer Registry, CancerCare Manitoba, Winnipeg, Manitoba, Canada; 7 Department of Family and Community Medicine, University of Toronto, Toronto, Ontario, Canada; 8 University Health Network, Toronto, Ontario, Canada; 9 Cancer Care Ontario, Toronto, Ontario, Canada; 10 Stanford University School of Medicine, Stanford, California, U.S.A.

## Abstract

Cancer care is complex and exists within the broader healthcare system. The CanIMPACT team sought to enhance primary cancer care capacity and improve integration between primary and cancer specialist care, focusing on breast cancer. In Canada, all medically-necessary healthcare is publicly funded but overseen at the provincial/territorial level. The CanIMPACT Administrative Health Data Group’s (AHDG) role was to describe inter-sectoral care across five Canadian provinces: British Columbia, Alberta, Manitoba, Ontario and Nova Scotia.

This paper describes the process used and challenges faced in creating four parallel administrative health datasets. We present the content of those datasets and population characteristics. We provide guidance for future research based on ‘lessons learned’.

The AHDG conducted population-based comparisons of care for breast cancer patients diagnosed from 2007-2011. We created parallel provincial datasets using knowledge from data inventories, our previous work, and ongoing bi-weekly conference calls. Common dataset creation plans (DCPs) ensured data comparability and documentation of data differences. In general, the process had to be flexible and iterative as our understanding of the data and needs of the broader team evolved.

Inter-sectoral data inconsistencies that we had to address occurred due to differences in: 1) healthcare systems, 2) data sources, 3) data elements and 4) variable definitions. Our parallel provincial datasets describe the breast cancer diagnostic, treatment and survivorship phases and address ten research objectives. Breast cancer patient demographics reflect inter-provincial general population differences. Across provinces, disease characteristics are similar but underlying health status and use of healthcare services differ.

Describing healthcare across Canadian jurisdictions assesses whether our provincial healthcare systems are delivering similar high quality, timely, accessible care to all of our citizens. We have provided a description of our experience in trying to achieve this goal and, for future use, we include a list of ‘lessons learned’ and a list of recommended steps for conducting this kind of work.

**Key Findings:**

The conduct of inter-sectoral research using linked administrative health data requires a committed team that is adequately resourced and has a set of clear, feasible objectives at the start.

Guiding principles include: maximization of sectoral participation by including single-jurisdiction expertise and making the most inclusive data decisions; use of living documents that track all data decisions and careful consideration about data quality and availability differences.

Inter-sectoral research requires a good understanding of the local healthcare system and other contextual issues for appropriate interpretation of observed differences.

## Introduction

The patient cancer experience is a trajectory, from understanding a new diagnosis, being involved in treatment decisions, dealing with the social and emotional effects of the diagnosis and, if all goes well, living life as a cancer survivor which can involve ongoing issues affecting quality of life. Cancer patients often have other health problems requiring them to manage their care across multiple health care settings. Consequently, cancer care changes by phase of disease, and necessarily exists within the broader health care system.

From both the patient and system perspectives, cancer care should be patient-centred, and integrated with the other health care a patient receives, to provide more effective, efficient, and acceptable care. But health care fragmentation is well documented and can be extreme (1—3). Family physicians, who are trained to have longstanding relationships with their patients and oversee the care of all their health conditions and preventive care, are one group of healthcare professionals that could help the system achieve whole-person, integrated care(1,4).

The Canadian Team to Improve Community-Based Cancer Care along the Continuum (CanIMPACT) was formed to ‘improve cancer care together’. Its overarching objectives are to enhance primary cancer care capacity and improve integration between primary and cancer specialist care along the cancer care continuum(5). We focussed on breast cancer care as an exemplar of what can be done to support these aims. Starting in September 2013, Phase 1 of the CanIMPACT program of research involved the conduct of foundational studies using a multimethod approach to inform the development of interventions in Phase 2, which began in the Spring 2016 and will be completed by April 2020. As part of Phase 1, the CanIMPACT Administrative Health Data Group (AHDG) undertook a description of breast cancer patients, their diagnostic process, their treatment and survivorship care across five Canadian provinces (British Columbia, Alberta, Manitoba, Ontario and Nova Scotia) to understand care and inform improvement efforts. Specifically, we conducted inter- and intra-provincial comparisons, focusing on aspects of care that may be influenced by primary care; and investigated whether vulnerable subgroups were at risk of sub-optimal access and outcomes. The purpose of this paper is to describe the process used and challenges faced in creating four parallel administrative health datasets, to present the content of those datasets and the characteristics of the resulting population-based provincial breast cancer cohorts, and to provide guidance for future such work based on ‘lessons learned’.

## Methods

### Context

In Canada, all medically-necessary health care is required to be publicly-funded, universal, comprehensive, and portable across provincial/territorial jurisdictions(6). Health care funding and delivery is the responsibility of the thirteen individual jurisdictions, and there are some differences in the actual health care by jurisdiction. Most outpatient physicians are “fee-for-service”, and primary care physicians play a “gate-keeper” role in access to specialty care. Cancer services other than surgery are usually offered within designated provincial cancer facilities.

### CanIMPACT Administrative Data Aims

The overall aim of the administrative health data component of the CanIMPACT research program was to conduct population-based comparisons of care for all breast cancer patients diagnosed from 2007 through 2011 (or latest available) in each of the five Canadian participating provinces. Provincial data sources included similarly structured population-based cancer registries that are linked to clinical and administrative health services data using individual encrypted health card numbers for research purposes. Details of these operations are found in provincial websites(7—11). All data sources used are stable and mature. They are linked routinely and used repeatedly in Canada to conduct health services research. The breast cancer outcomes we studied included detection method (screened or symptomatic), diagnostic interval length, use of adjuvant chemotherapy, chemotherapy toxicity and attendant use of emergency departments, survivorship care guideline adherence and use of primary care and oncology care across the continuum from diagnosis through survivorship. Comparisons were made across provinces and regionally within provinces, and by vulnerability indicators: age at diagnosis, rurality, area-level socioeconomic status, area-level immigration status, and comorbid disease status.

### Data Management Approach

The CanIMPACT Administrative Health Data Group (AHDG) has twenty members, with combined expertise in primary care, surgery, medical oncology, epidemiology, biostatistics, data processing, economics, and cancer registries and it includes three patient representatives The AHDG membership includes a lead from each province with expertise in the use of the their provincial health administrative data to provide informed data processing and interpretation and ensure adherence to provincial security/ privacy rules. Our research methods were informed by the collective research experience from the members of our team including the conduct of similar studies using administrative databases in individual provinces(12—21). Analyses were conducted separately at designated research centres in each province using similar strategies guided by a common data processing and analysis plan. Knowledge of each province’s policy environment and health care structure was also required in order to interpret study findings. This knowledge was provided by AHDG members and the wider CanIMPACT team.

The lead and some other AHDG members with expertise in their provincial data and/or analysis constituted a core working group that communicated regularly, with patient advisors and other AHDG members participating whenever possible. Bi-weekly conference calls have been the core communication strategy of the AHDG with more than sixty documented calls over a three-year period. Ongoing email communications and a number of face-to-face meetings attended by key members complete our communication strategy. Through this communication strategy we refined and operationalized the research objectives, identified and processed data elements using standardized definitions, and developed ten study objectives and analysis plans for publication(22—24).

### AHDG Activities

In Canada, federal and provincial data protection laws provide insufficient guidance regarding data release for research purposes, leading to inconsistent inter-provincial data sharing policies across provinces(25). Although there have been some instances in which a country-wide research dataset has been created, the effort involved is considerable and was beyond the scope and time constraints of our project. We therefore produced separate, parallel project datasets and analogous analysis plans across provinces to meet the group’s objectives. These datasets would contain information on the demographic, clinical and healthcare utilization of a cancer patient cohort from one to two years prior to diagnosis through up to ten years post-treatment. The tasks involved in creating these parallel project datasets included understanding overlap and gaps in file and data availability and determining common variable definitions. Creation of these data resources was an iterative process of refining research objectives and analysis plans as feasibility issues related to data quality and/or availability were identified.

Our understanding of the data nuances evolved throughout the course of the study. As part of the grant development and continuing after funding was received in April 2013, before data were available, decisions about variable capture relied on team members’ knowledge, provision of variable frequencies from previously cut datasets and on country-wide reporting of data by government agencies. As a first step in the research process, we produced an inventory of data sources and potentially relevant data elements in each province. This allowed us to assess, at a high level, whether the provinces involved had access to similar data and identify obvious limitations. The initial data sources considered included the provincial cancer registry, provincial health insurance plan client registry, hospital discharge abstracts, outpatient physician service claims, hospital outpatient services including emergency services, continuing care data, mental health services data, elderly prescription drug data and/or population-wide prescription drug data, cancer treatment data, and immigration data. With the exception of immigration data which is probabilistically linked, all other data are deterministically linked at the individual level using provincial health insurance numbers. This inventory revealed that some provinces did not have access to hospital outpatient services, population-wide prescription data, and/or individual-level immigration data. These limitations informed the selection of our first draft set of key data elements.

Some of the key AHDG group members met in person for two days in September 2013 to further critique our experiences, present methodologies we had developed in our respective single-province studies, and compare findings. We discussed specific aspects of complex variable definitions, problems with missing data, and issues about data validity. Shortly after this workshop we started regular conference calls to continue the discussion.

We gathered further information about each key data element including: its description/definition, data source, coverage years and any relevant background information. One of the study investigators (MW) summarized this information and assessed feasibility for inclusion. Examples of issues that arose at this stage included age being defined based on month and year only in one province, area-level immigration data were only available in two provinces, and one province used less precise diagnostic codes in its physician billing data.

At this point we began to create four dataset creation plans (DCPs) for each phase of care we planned to study: baseline/diagnosis phase; treatment phase; and survivorship phase. We used a DCP template from ICES in Ontario which requires documentation of the study personnel, edition changes, study goals and objectives, datasets to be used, study timeframe and key dates, study variables and analysis plan. These living documents provided a road map for data programmer-analysts in each province and serve as reference documents for data dictionary development, key decisions, and our knowledge about inter-provincial data differences. Development of the DCPs focused our thinking on the details needed to address the phase-of-care-specific research questions. These details included specific inclusion/exclusion criteria needed for each phase, defining the diagnosis period and follow-up time, details involved in data processing and variable definitions including ensuring data comparability across phases to facilitate longitudinal analyses, descriptive statistics, and statistical modeling. Our discussions and decisions were informed by team members’ previous work, as mentioned above. For instance, determination of the diagnostic interval(12—15), chemotherapy toxicity codes(16,17), and censoring decisions during survivorship(18—21) were imports from this previous work. We also took advice from a national cancer quality of care reporting agency regarding the best choice for characterizing area-level immigration status, rurality and area-level socioeconomic status(26).

At the one-year point and in preparation for a full-team face-to-face meeting in October 2014, we took a step back and developed a study framework that mapped our key data elements onto the dimensions of access and quality defined by Andersen(27) and the WHO(28). These dimensions included: coordinated care, effective care, efficient care, accessible care, acceptable/patient-centered care, equitable care and safe care. The framework considered the three phases of the cancer care continuum we were studying (diagnosis, treatment and survivorship) and the relevant, responsible healthcare providers. After having initially taken a broad perspective on what we could accomplish, this exercise, with the help of the full CanIMPACT team, helped us refocus on the big issues around coordinated high quality cancer care and further refine our plans.

We presented our preliminary findings at the CanIMPACT Consultative Workshop, held in March 2016 as our contribution to the CanIMPACT Phase 1 goals. The workshop included all members of the CanIMPACT team and others, including knowledge users and patients with 74 attendees in all. The outcome of that workshop was a decision on a direction for the intervention to be conducted in Phase 2 of the CanIMPACT study(29). Since then we have completed data processing and analyses addressing ten research objectives for publication. 

## Results

### Study Process Experiences

Overall, we found that frequent, meaningful communications and the commitment of the team members were key to our success and we had to allow for flexibility in the study process and analytic details as further data understanding occurred.

Inter-provincial data inconsistencies can be summarized across four dimensions: 1) system practice 2) data source 3) raw data element, and 4) variable definition. An example of system practice level variation was the “cancer diagnostic assessment program”, which is an Ontario initiative that oversees the diagnostic process using a multidisciplinary team approach(30). No other province had a similar program so the impact of such programs was dropped from our objectives. Instead, the existence of this program serves as a contextual element that informs our interpretation of our study findings. Another potential system-level source of variation was the quality of claims data by method of physician payment (fee for service versus alternative payment plans). For instance, 5% of Ontario specialists and 50% of Ontario primary care physicians are remunerated under alternative payment plans. However, they are required to shadow bill and are often given cash incentives for doing so. Completeness and accuracy have been shown to be high for both payment forms in a recent study conducted in the Province of Alberta(31). We mitigated potential claims errors by: 1) emphasizing visit counts whenever possible (which only require the existence of a claim on a particular day); 2) using hospitalization data to assign surgery type; 3) grouping all imaging into a single variable; and 4) using established claims-based chronic disease algorithms which require more than one occurrence of a diagnostic code for assignment of disease status(32). At the data source level, two databases were not available to all provinces. Immigration, Refugees and Citizenship Canada’s permanent resident data, which contains demographic information for every landed immigrant, were only available in British Columbia and Ontario. We are reporting on the immigrant experience in those two provinces. National Ambulatory Care Reporting System data that standardizes reporting on emergency department visits across Canada were available only in Ontario and Alberta. Fortunately, Nova Scotia and Manitoba had strategies for identifying emergency room visits in the absence of the National Ambulatory Reporting System data that they shared with British Columbia. Data quality and coverage is particularly high for the provincial cancer registries which meet the certification quality standards required for North American reporting (except Ontario)(33) and WHO reporting(34). Quality is also high for hospital inpatient reporting, which is required for all Canadian jurisdictions using standardized data(35). The number of breast cancer patients leaving their home province is likely to be low since only 6.3% of internal migrants in Canada are over 65 years of age(36), thus large losses to follow up are not a concern. Double-counting across provinces is not an issue either since we only studied incident cases. At the raw data element level, an example of data inconsistency was the “date of death” variable. All provinces had date/month/year information except for British Columbia, which only had month/year so survival data are computed at that level of precision.

Similar data resources could contain fundamental differences that were only revealed once we were producing detailed data processing plans. For instance, in Canada we can use the “Postal Code Conversion File (PCCF)”(37) created by Statistics Canada to assign people to census areas based on their postal code. The PCCF is then able to identify many area-level data items relevant to that person from the census, such as socioeconomic status. But there are many consecutive versions of the PCCF that contain subtle variable definition differences or even different data. As it turned out, the PCCF version available in Ontario did not contain the area-level immigration tertile variable which we used for area-level immigration status, and the PCCF version available in British Columbia did not contain a geocode that was required to create a deprivation index.

Data processing to create comparable data items varied based not only on variations in data structure and availability but also on variations in the structure of the respective provincial healthcare systems. For instance, whereas all mammography screening occurs and is documented in organized programs in the other provinces, in Alberta and Ontario, screening mammography can occur outside the organized screening programs, requiring the application of algorithms to other databases to identify those patients(13,15).

Results interpretation also had to consider the provincial context. For instance, screening rate differences had to be interpreted with an understanding of screening age eligibility variations over time and across provinces. Health system structural differences could explain inter-provincial variation. For instance, in Nova Scotia, the diagnostic interval was similar for screened and symptomatic patients because of the centralized nature of their diagnostic services. In other provinces, the symptomatic patients waited longer for a diagnosis.

Even with population-wide data sources in a common cancer, sample size concerns dictated some decisions. Our aim to study the effect of a breast cancer diagnosis on chronic disease care was limited by small numbers of documented chronic disease in the smaller provinces and by incomplete data (see Supplementary Appendix 1 for details). We had to include cases as far back as 2007 to ensure enough numbers in the smaller provinces because we also had to end our recruitment (2011 diagnoses) with enough time to study the survivorship phase.

We used the following principles in the presence of data differences:

1. *Maximize the number of provinces contributing by making the most inclusive choice.* For instance, in Nova Scotia, chemotherapy data is known to be incomplete, but information about consultations with medical oncologists is available. Based on patterns of visits to medical oncology, we determined who received chemotherapy and the start date for chemotherapy receipt. We were, however, unable to determine a chemotherapy end date in Nova Scotia so an average chemotherapy treatment duration was used instead.
2. *Use previously-developed methods and definitions whenever possible.* For example, British Columbia did not have access to emergency room data but Manitoba had an algorithm previously developed and validated using hospital discharge data to find emergency room visits that British Columbia adopted for the study.
3. *Track differences in key study variables between provinces in the DCPs to ensure this is considered when interpreting results.* For example, stage information was collected differently across provinces, with variable use of clinic-assigned stage and use of cancer registrars to assign collaborative stage(38), requiring the use of only stage groups (I-IV) for consistency.
4. *Track study variable quality in the DCPs to ensure this is considered when interpreting results.* For example, area-level SES assignment depends on mapping census dissemination areas to postal codes. The error rate on this mapping is high in rural areas and needed to be considered when comparing SES effects.

### Study Data Sources and Variables

Supplementary Appendix 1 provides details on the datasets, including study variable definitions, with source references when applicable, data sources, inter-provincial definitional differences and data availability. The main data sources we used included similarly structured cancer registry, census area-level demographic data and provincial administrative databases, including physician claims, ambulatory care and inpatient hospital data. Our population-based datasets contain information on patient socio-demographics, baseline health status, breast cancer disease characteristics, health care use across the cancer care continuum, the diagnostic method and timeliness, initial treatment and waits for chemotherapy, treatment toxicity, survivorship care guideline adherence, and survival. The methods used for data capture of all data sources used were stable across the period of the study. In the Supplementary Appendix 1 we have also documented our attempts to identify chronic disease cohorts and chronic and preventive care.

### Cohort Description

The datasets contain information on all histologically-confirmed breast cancer patients (ICD 174) diagnosed in these provinces for the years listed in Table 1 as captured in our provincial, population-based cancer registries. The size and demographics of the study cohorts are described in Table 1. The results reflect known inter-provincial differences in general population demographics(39). The median age (IQR) was 61 (51-72) in British Columbia, 62 (52-72) in Manitoba, 60 (50-71) in Ontario, and 62 (52-72) in Nova Scotia. Median age was not available for Alberta but it included more patients in the 40-49 group and correspondingly fewer in the >74 group. Area-level socioeconomic status patterns were similar across provinces but with slightly fewer in the lowest income quintile in Manitoba and Ontario. In contrast, the pattern for area-level material deprivation for the three provinces reporting shows larger differences, with 49% of Ontario patients in the two least deprived groups compared to 38% in Manitoba and 28% in Nova Scotia. Conversely, 35% of Nova Scotia patients fell in the most deprived quintile for that province. The difference in the results of these two socioeconomic variables is explained by the fact that the income quintile boundaries were set using the provincial distribution while the deprivation quintile boundaries were set using the country-wide distribution. Therefore, larger differences for deprivation compared to income are due to inter-provincial SES differences. There are more immigrants in British Columbia than the other two provinces reporting immigration tertile and larger urban populations in British Columbia and Ontario.

**Table 1: Demographic characteristics of the CanIMPACT provincial breast cancer cohorts (%):** BC British Columbia; AB Alberta; MB Manitoba; ON Ontario; NS Nova Scotia NA Not available

	BC	AB	MB	ON	NS
Overall N	14,198	12,373	4,216	46,966	3,802
Diagnosis years	2007-2011	2004-2010	2007-2012	2007-2012	2007-2012
**Age**					
<40	4.1	5.3	4.5	6.0	3.8
40-49	16.6	21.5	15.1	16.4	16.3
50-59	24.0	48.9	23.9	25.1	22.2
60-69	25.6		25.6	25.0	27.0
70-74	9.6	8.5	9.2	9.4	10.0
>74	20.1	15.8	21.7	18.1	20.8
**Neighbourhood Income Quintile (SES)**					
1-Lowest	18.6	18.2	16.2	17.3	18.2
2	19.7	19.7	20.8	19.4	21.0
3	19.3	20.6	20.2	19.4	19.8
4	19.5	19.3	21.7	21.4	21.0
5-Highest	20.9	21.8	20.9	22.2	19.8
Unknown	2.0	0.4	0.2	0.3	0.2
**Deprivation Index Quintile**					
5 - Most deprived	NA	NA	18.2	12.3	34.7
4			21.0	16.4	21.1
3			19.3	20.7	14.9
2			18.2	23.1	14.8
1			19.8	26.2	13.4
Unknown			3.5	1.2	1.1
**Immigration Status (Urban only) N**	12,186		2,996		2,447
Lowest Tertile	51.8	NA	81.1	NA	98.5
Middle Tertile	30.2		17.3		0.8
Highest Tertile	15.7		0.9		0.0
Unknown	2.4		0.7		0.7
**Urban/Rural Residence**					
Urban	85.8	78.7	71.1	87.7	64.4
Rural	2.4	21.3	2.5	5.0	35.6
Rural-remote	4.4		9.2	4.7	
Rural-very remote	6.5		17.0	2.5	
Unknown / rural-unknown	0.8	0	0.2	0	0

Table 2 describes the disease characteristics and comorbid illness burden of these provincial breast cancer cohorts. Three provinces (Alberta, Manitoba, Nova Scotia) had almost complete information on breast cancer stage. The stage distributions for these three provinces are similar (if we exclude the carcinoma in situ group in Alberta to mimic the other cohorts) except that the Stage IV group is smaller in Alberta at 3.8% compared to Manitoba and Nova Scotia at 6.1%. Histologic grade distributions for provinces with reasonable completeness were similar, with the largest difference being a 6% lower rate of poorly differentiated cancers in Manitoba compared to Alberta and Nova Scotia. This information was missing for 50% of Ontario patients. Comorbid illness counts, as measured by the Johns Hopkins Adjusted Clinical Group (ACG) system(40) revealed a lower comorbid illness burden in British Columbia than in the other provinces with 32% having a 0-3 count. Ontario patients also had more patients in this group at 26.4% compared to Manitoba at 23.6% and Nova Scotia at 21.9%. Although not directly comparable, we have Charlson comorbidity scores(41,42) for Alberta, with 72.4% having a score of 0 comorbidities on this scale, 19% with 1 and 8.6% with more than one. More patients in Nova Scotia were high users of the health care system at 18.3% compared to 13.6% in British Columbia, 16.9% in Manitoba and 16.5% in Ontario.

**Table 2: Demographic characteristics of the CanIMPACT provincial breast cancer cohorts (%):** BC British Columbia; AB Alberta; MB Manitoba; ON Ontario; NS Nova Scotia ACG Adjusted Clinical Groups; ADG Adjusted Diagnostic Groups; RUB Resource Utilization Band NA Not available

	BC	AB	MB	ON	NS
Overall N	14,198	12,373	4,216	46,966	3,802
Diagnosis years	2007-2011	2004-2010	2007-2012	2007-2012	2007-2012
Stage					
In Situ (Alberta Only)	NA	12.8	NA	NA	NA
I	42.0	38.8	40.5	37.2	44.2
II	32.0	30.9	37.5	35.4	33.4
III	12.7	12.0	14.3	13.0	12.6
IV	4.2	3.3	6.1	4.4	6.1
Unknown	9.0	3.1	1.6	10.0	3.7
Histologic Grade					
Well differentiated	20.5	19.1	19.2	10.9	16.6
Moderately differentiated	37.7	40.2	42.9	22.7	38.0
Poorly or undifferentiated	31.7	35.6	29.4	15.9	35.8
Unknown	10.2	5.1	8.4	50.4	9.6
Co-morbidity (ACG System-ADGs)					
0-3 ADGs	32.1	NA	23.6	26.4	21.9
4-5 ADGs	24.3		22.5	22.8	20.9
6-7 ADGs	19.7		20.4	21.1	20.2
8-9 ADGs	12.9		16.2	15.0	16.7
10+ ADGs	11.0		17.3	14.8	20.3
Co-morbidity (ACG System-RUBs)					
0 (no or invalid diagnosis)	5.8	NA	3.6	4.8	4.0
1 (healthy user)	2.4		3.8	2.7	2.1
2 (low)	12.5		10.7	11.0	9.0
3 (moderate)	60.2		58.8	58.5	58.9
4 (high)	13.6		16.9	16.5	18.3
5 (very high)	5.5		6.2	6.5	7.6

## Discussion

### Effective Practices

We have described a cross-province collaboration involving a strong, committed team of researchers, knowledge users and patients who worked together to describe and assess differences in inter-sectoral breast cancer care. The practices we adopted that proved effective included: concurrent data definition and development of detailed analysis plans across jurisdictions; frequent, structured communication within a core group; scheduled “check-ins” with the full group at key points in development of the research plans and utilization of previous study definitions and methods whenever possible. These strategies led to the creation of four comparable datasets that are allowing the reporting of breast cancer care and outcome patterns across Canada.

A critical component to maintaining good organization and documentation management, both essential given the complexity of the endeavor, was designating one research associate (LJ) to be responsible for keeping the dataset creation plans up-to-date based on decisions made during our conference calls and meetings. This associate also fielded all clarification questions from the provinces, forwarding them to appropriate investigators as needed. She kept track of action items and worked with the group’s co-chairs to set conference call agendas. Additionally, to further clarify the data requests and analyses, she created templates for data tables needed with the agreed upon demographic, clinical and healthcare utilization factors specific to each data analysis plan.

The regular conference calls were critical to the successful completion of the analyses, and we continue our regular conference calls as manuscripts are being developed. These calls and ongoing email correspondence help with manuscript refinement and reconciliation of any further data inconsistencies that become evident as we are reporting on the results.

### Challenges

Data privacy and ethics board requirements differed across provinces with regard to the amount of study information needed for approval. Data access processes varied across provinces, with it taking longer to receive a de-identified dataset in some provinces than in others. This complicated efforts to perform analyses in parallel and in some cases reduced the timeliness with which final results were made available for dissemination. Importantly, the process was most straightforward in the provinces with centralized linked data repositories collated for research purposes. Challenges varied depending on the number of jurisdictions involved. In the current study we were able to enroll five of the thirteen provincial/territorial jurisdictions in Canada.

The research plan evolved with the operationalization of our initial high-level objectives and increasing understanding about data availability and other feasibility issues. We initially thought we could use existing breast cancer cohort databases but since our data elements were different, or provincial access rules required that databases be recreated from scratch, these pre-existing datasets were useful only for some preliminary data analyses but not for the final work. Adding new objectives mid-course led to fairly large changes to the dataset development and analysis plans. Specifically, we added tumour markers, we added an objective to assess the association between time to chemotherapy and survival, and the result of one of the full-team face to face meetings was a decision to include quality of chronic and preventive disease indicators at baseline and during survivorship. These additions were not as successful as our original goals; only one province had near-complete tumour marker data, we were unable to run the survival analyses due to budget constraints, and chronic disease indicator exercise was subsequently reduced to a focus on chronic obstructive pulmonary disease (COPD) and diabetes care due to small numbers in the smaller provinces.

Budgeting was difficult due to the complexity, scope and changing nature of the project and due to variation in dataset readiness and available provincial infrastructure support. The planning phase was much longer than expected leading to the loss of the study coordinator before the process was complete due to budgetary constraints.

Personnel availability can change over the course of a large study which can disrupt continuity. In this study, the Alberta lead moved to the United States and although she continues to be actively involved and one of her colleagues fortunately stepped in to continue to provide data access, in the end, we were only able to include Alberta on some of the diagnostic phase analyses.

### Other Similar Work

Other researchers have conducted similar studies of cross-jurisdictional healthcare quality and access.(43) Within Canada, for instance, Barbera and colleagues created parallel datasets to study quality indicators of palliative care across four Canadian provinces(44). With regard to the effort involved, they concluded that conducting inter-provincial comparisons in the absence of data sharing agreements makes ongoing surveillance of palliative care quality indicators unlikely. Inter-country studies of healthcare patterns have also been conducted, such as that by Gigli and colleagues looking at colorectal cancer care in Italy and the United States(45) and Warren and colleagues who compared end of life care in Ontario and the United States(46xref>). As Lipscomb points out, these studies were possible because the jurisdictions involved could link established cancer registries to administrative healthcare data longitudinally(43).

### Lessons Learned

Based on our experiences with this project, we have drafted a checklist of principles and processes that could be used for future cross jurisdictional research that are provided in Box 1 and a suggested checklist for undergoing similar future studies in Box 2.

Box 1. **Lessons Learned**

1. The project team should include expertise in the medical issue under study, the local healthcare systems, study design and analysis using the local routinely collected health data. The study should employ an overall coordinator. Data programmer/analyst(s) for each jurisdiction should be involved in every study meeting.
2. Develop core, living dataset creation plans including all documentation on data differences and validity assessments.
3. Generate table templates for results in the form of side-by-side spreadsheets with a column for each jurisdiction.
4. Consider feasibility based on amount of missing data and expected cohort sizes.
5. Conduct systematic assessment of each variable under consideration with highly informed team members regarding local culture/policies/uses as well as data quality.
6. Use variables that have been previously developed and use standard variable definitions whenever possible.
7. Consider for each variable whether there is a common data source type (e.g., physician billings, hospitalization data) available to each jurisdiction. If one or more jurisdictions have access to more than one of the potential data sources, consider checking comparability by conducting sensitivity analyses.
8. Consider the use of standardized computer programs for the analyses and, to the extent it is feasible, data processing.
9. Set up a centralized document archive to be overseen by the study coordinator and make it easily accessible to all members of the team. Actively promote its use and make sure key documents are kept separate from background documents. Find ways to make sure you do not redo or lose work/knowledge by hot linking documents containing clinical insights, data analysis plans and validation analyses to their related working documents (such as DCPs and draft manuscripts). Ensure data custodian reporting and acknowledgement requirements are easily accessible and up to date in this centralized archive.

Box 2. **Recommended Steps to Conduct an Inter-Jurisdictional Administrative Data Study**

1. Create a team that includes at least two core members in each jurisdiction to ensure continuity.
2. Set clear objectives based on an established conceptual framework with a commitment from all involved that the scope / objectives are final.
3. Create an initial list of the study variables.
4. Conduct an initial assessment of data completeness, availability and comparability.
5. Reconcile objectives and study variable list based on 4).
6. Reconcile any differences in ability to apply inclusion/exclusion criteria.
7. Obtain data access including research ethics board approvals.
8. List the planned manuscripts with lead authors and writing team membership assigned and draft analysis plans with dummy tables and figures for each manuscript included.
9. Reconcile objectives, study variable list, data processing and analysis plans based on 8).
10. Run initial data processing.
11. Run second assessment of data completeness, availability and comparability including planned validation analyses.
12. Reconcile manuscript plans including objectives, study variable list, data processing plans and analysis plans based on 11).
13. Carefully adhere to data custodian results reporting and acknowledgement requirements in each jurisdiction.
14. Develop a knowledge dissemination strategy that includes presentations at academic conferences and also targets relevant non-academic stakeholders.

### Conclusions and Future Directions

Documenting differences in health care across Canadian jurisdictions is crucial for understanding whether our provincial health care systems are delivering similar high quality, timely, accessible care to all of our citizens as mandated by the Canada Health Act(6). Restricting such description to single data sources cannot provide a comprehensive picture of health care delivery, so cross-data source inter-sectoral linkage projects such as this are an important evolution toward our ability to study the complete health care experience across the thirteen jurisdictional health care systems in Canada. The development of parallel linked datasets across national or international jurisdictions can also inform our understanding of whether factors associated with access and quality such as vulnerable group status are universal or healthcare context specific. We note that future use of parallel datasets such as ours could be subjected to summary data meta-analysis for pooled effects(47) allowing the quantitative assessment of generalizability of observed effects over multiple jurisdictions in addition to summary effect estimates.

Data resources and availability are ever-changing based on changes in the health care system, health care informatics, and privacy and research ethics and legislation. In future, we hope that the conduct of projects such as ours will become more streamlined with regard to data access and common data elements. The SEER-Medicare linked database in the United States has been used for many years to conduct cross-jurisdictional cancer-related health services research(48,49). The development of distributed data networks, in which similarly structured parallel datasets are subjected to the same analytic code, is a welcome development(47). The Canadian Network for Observational Drug Effect Studies (CNODES) is a Canadian example(50) that is supported by the Canadian Institutes for Health Research and is filling the need for a large data source to study rare adverse drug events.

The creation of a single national healthcare data source containing the level of detail that we were able to capture is a much greater challenge - especially in a country such as Canada in which healthcare is the responsibility of the provinces. Barriers to accessing and analyzing health information in Canada have been described(51). Recommendations for overcoming those barriers include the need for harmonized ethics approaches, legislation, policies and procedures for accessing and sharing data, the existence of strong federal-provincial-territorial partnerships and support, and more standardized data across the commonly-used data sources(51). Lipscomb specifies the need to include partnerships with between government agencies, professional organizations, provider organizations and researchers and cautions that that feasibility for building and maintaining such a resource would be very challenging(43).

We faced many hurdles in creating parallel datasets for a single study and we thought it important to report the learning from this effort for future research. In two of the provinces in this study the work was done at longstanding provincial data centers with robust infrastructure and data experience. It was clear to us that the existence of those centers simplified a lot of the data access and processing that was required in comparison to the provinces without those resources. If such resources could exist in each of our provinces and territories it would greatly enhance our ability to study healthcare delivery both at the provincial and national levels. We have also learned that local knowledge about health system structure, practices and policies is crucial to data use and interpretation. Local knowledge will always need to be an integral part of the use of such data, even if we are successful in creating a national resource. 

## Acknowledgments

In addition to the authors, the membership of the CanIMPACT Administrative Health Data Group who undertook this work include: Natalie Biswanger, CancerCare Manitoba, Winnipeg, Manitoba; Dongdong Li, BC Cancer Agency, Vancouver, British Columbia; Aisha Lofters, University of Toronto, Toronto, Ontario; Sharon Matthias, CanIMPACT Patient Advisory Committee, Alberta; Nicole Mittmann, Cancer Care Ontario, Toronto, Ontario; Rahim Moineddin, University of Toronto, Toronto, Ontario; Geoff Porter, Dalhousie University, Halifax, Nova Scotia; Dawn Powell, CanIMPACT Patient Advisory Committee, Ontario; Donna Turner, CancerCare Manitoba, Winnipeg, Manitoba; Robin Urquhart, Dalhousie University, Halifax, Nova Scotia; Bonnie Vick, CanIMPACT Patient Advisory Committee, Saskatchewan; Yan Yuan, University of Alberta, Edmonton, Alberta

The authors would also thank Emma Shu, Marlo Whitehead, and Yan Zhang for conducting data processing and statistical analyses.

This study was funded by the Canadian Institutes of Health Research (grant # 128272). The opinions, results and conclusions reported in this paper are those of the authors and are independent from the funder. This study is supported by ICES, which is funded by an annual grant from the Ontario Ministry of Health and Long-Term Care (MOHLTC). No endorsement by ICES or the Ontario MOHLTC is intended or should be inferred. Parts of this material are based on data and information provided by Cancer Care Ontario (CCO). The opinions, results, views, and conclusions reported in this paper are those of the authors and do not necessarily reflect those of CCO. No endorsement by CCO is intended or should be inferred. Parts of this material are based on data and information compiled and provided by the Canadian Institute for Health Information (CIHI). However, the analyses, conclusions, opinions and statements expressed herein are those of the author, and not necessarily those of CIHI. We gratefully acknowledge CancerCare Manitoba for their on-going support and Manitoba Health for the provision of data. The results and conclusions presented are those of the authors. No official endorsement by Manitoba Health is intended or should be inferred. Nova Scotia data were provided by Health Data Nova Scotia and the Nova Scotia Department of Health and Wellness, however, the observations and opinions expressed are those of the authors and do not represent those of either Health Data Nova Scotia or the Department of Health and Wellness. Data for this study were also provided by Population Data BC and the BC Cancer Agency. All inferences, opinions, and conclusions drawn in this study are those of the authors, and do not reflect the opinions or policies of the BC Data Steward(s)(52—56).

## Supplementary Appendix

Supplementary Appendix 1 provides details on the datasets, including study variable definitions, data sources, inter-provincial definitional differences and data availability.

## Abbreviations

ACG	Adjusted Clinical Groups
ADG	Adjusted Diagnostic Groups
AHDG	Administrative Health Data Group
CanIMPACT	Canadian Team to Improve Community-Based Cancer Care Along the Continuum
CNODES	Canadian Network for Observational Drug Effect Studies
COPD	Chronic Obstructive Pulmonary Disease
DCP	Dataset Creation Plan
ICD	International Classification of Diseases
IQR	Interquartile Range
PCCF	Postal Code Conversion File
RUB	Resource Utilization Band
WHO	World Health Organization
SES	Socioeconomic Status

## References

[1] World Health Organization. The World Health Report 2008 Primary health care: now more than ever. Geneva, World Health Organization; 2008 [cited 2017 November 29]. 110p. Available from: www.who.int/whr/2008/en/.

[2] United States, The National Academies, Institute of Medicine. Delivering high quality cancer care: charting a new course for a system in crisis. Washington, DC: The National Academies Press; 2013. 24872984

[3] Canadian Partnership Against Cancer. Examining disparities in cancer control: a system performance special focus report. Toronto, Canadian Partnership Against Cancer; 2014 [cited 2017 November 29]. 88p. Available from: www.cancerview.ca/systemperformancereport

[4] Rubin G, Berendsen A, Crawford SM, Dommett R, Earle C, Emery J, et al The expanding role of primary care in cancer control. Lancet Oncol. 2015;16(12):1231-1272. 10.1016/S1470-2045(15)00205-3 26431866

[5] Grunfeld E. It takes a team. CanIMPACT: Canadian Team to Improve Community-Based Cancer Care along the Continuum. 2016;62(10):781-782. PMC5063759 27737968

[6] Madore O. The Canada Health Act: overview and options. Ottawa, Ontario: Ottawa Library of Parliament, Parliamentary Research Branch; 2003.

[7] Population Data BC. [Internet]. Services for researchers. [cited 2018 June 11];[about 3 screens]. Available from: https://www.popdata.bc.ca/researchers

[8] Alberta Health Services. [Internet]. Research and innovation at Alberta Health Services. [cited 2018 June 11];[about 2 screens]. Available from: https://www.albertahealthservices.ca/research/research.aspx

[9] Manitoba Health. [Internet]. Health Information Privacy Committee. [cited 2018 June 11];[about 1 screen]. Available from: http://www.gov.mb.ca/health/hipc/

[10] ICES. [Internet]. ICES data. [cited 2018 June 11];[ about 4 screens]. Available from: https://www.ices.on.ca/Data-and-Privacy/ICES-data

[11] Health Data Nova Scotia [Internet]. Our services. [cited 2018 June 11];[about 3 screens]. Available from: https://medicine.dal.ca/departments/department-sites/community-health/research/hdns/services.html

[12] Sikdar K, Dickinson JA, Winget M. Factors associated with mode of colorectal cancer detection and time to diagnosis: a population level study. BMC Health Services Research, 2017;17:7. 10.1186/s12913-016-1944-y PMC5376684 28056946

[13] Yuan Y, Li M, Yang J, Winget M. Using administrative data to estimate time to breast cancer diagnosis and percent of screen-detected breast cancers – A validation study in Alberta, Canada. European Journal of Cancer Care. 2015;24:367-375. 10.1111/ecc.12277 25521706

[14] Yuan Y, M Li, Yang J, Elliot T, Dabbs K, Dickinson JA, et al Factors related to breast cancer detection mode and time to diagnosis in Alberta, Canada: a population-based retrospective cohort study. BMC Health Services Research. Published online 19 2 2016 10.1186/s12913-016-1303-z, 2016 PMC4759735 26892589

[15] Jiang L, Gilbert J, Langley H, Moineddin R, Groome PA. Effect of Specialized Diagnostic Assessment Units on the Time to Diagnosis in Screen-detected Breast Cancer Patients. Br J Cancer 2015; 112(11):1744-1750. 10.1038/bjc.2015.147 25942395 PMC4647239

[16] Bastedo SJ, Krzyzanowska M, Moineddin R, Yun L, Enright KA, Grunfeld G. A population-based assessment of primary care visits during adjuvant chemotherapy for breast cancer. Curr Oncol. 2017;24(2):90-94. 10.3747/co.24.3431 28490922 PMC5407871

[17] Krzyzanowska MK, Enright K, Moineddin R, Yun L, Powis M, Ghannam M, et al Can chemotherapy-related acute care visits be accurately identified in administrative data? J Oncol Pract 20 11 2017: JOP2017023697. doi: 10.1200/JOP.2017.023697. [Epub ahead of print]. 29155611

[18] Grunfeld E, Hodgson DC, Del Giudice ME, Moineddin R. Population-based longitudinal study of follow-up care for breast cancer survivors. J Oncol Pract 2010;6(4):174-181. 10.1200/jop.200009 21037867 PMC2900866

[19] McBride ML, Lorenzi MF, Page J, Broemeling A-M, Spinelli JJ, Goddard K, et al Patterns of physician follow-up among young cancer survivors. Cdn Fam Phys. 2011;57(12):e482-e490. PMC3237536 22170210

[20] Lorenzi M, Xie L, Rogers P, Pritchard S, Goddard K, McBride ML. Hospital-related morbidity among childhood cancer survivors in British Columbia, Canada: report of the Childhood Adolescent, Young Adult Cancer Survivors (CAYACS) program. Int J Cancer 2011;127(7):1624-1631. 10.1002/ijc.25751 21280033

[21] Urquhart R, Folkes A, Porter G, Kendell C, Cox M, Dewar R, et al Population-based longitudinal study of follow-up care for colorectal cancer patients in Nova Scotia. J Oncol Pract 2012;8(4):246-252. 10.1200/jop.2011.000491 23180991 PMC3396823

[22] Jiang L, Lofters A, Moineddin R, Decker K, Groome P, Kendell C, et al Primary care physician use across the breast cancer care continuum: CanIMPACT study using Canadian administrative data. Cdn Fam Phys 2016;62:e589-e598 PMC5063785 27737994

[23] Kendell C, Decker KM, Groome PA, McBride M, Jiang L, Krzyzanowska MK, et al, for the CanIMPACT Team. Utilization of physician services during the survivorship phase: a multi-province study of women diagnosed with breast cancer. Curr Oncol 2017;24(2):81-89. 10.3747/co.24.3454 28490921 PMC5407870

[24] O’Brien MA, Carroll JC, Manca DP, Miedema B, Groome PA, Makuwaza MA, et al Multi-gene expression profile testing in breast cancer: is there a role for family physicians? Curr Oncol 2017;24(2):95-102. 10.3747/co.24.3457 28490923 PMC5407872

[25] Willison DJ. Data protection and the promotion of health research: if the laws are not the problem, then what is? Healthcare Policy 2007;2(3):39-43 10.12927/hcpol.2007.18699 19305716 PMC2585460

[26] Canadian Partnership Against Cancer. [Internet]. Examining disparities in cancer control: a system performance special focus report. 2 2014 [cited 2018 June 10];[pdf] Available from: https://content.cancerview.ca/download/cv/quality_and_planning/system_performance/documents/spexamdispreportpdf?attachment=0

[27] Andersen RM. Revisiting the behavioral model and access to medical care: does it matter? J Hlth Soc Behav 1995;36(1):1-10. 10.2307/2137284 7738325

[28] World Health Organization. [Internet]. Quality of care: a process for making strategic choices in health systems. Geneva, World Health Organization; 2006 [cited 2017 November 29];. 50p. Available from: www.who.int/management/quality/assurance/QualityCare

[29] Grunfeld E, Petrovic B, for the CanIMPACT investigators. Consultative workshop proceedings of the Canadian Team to Improve Community-Based Cancer Care Along the Continuum. Curr Oncol. 2017;24(2):135-140. 10.3747/co.24.3436

[30] Ontario Breast Screening Program [Internet]. Toronto: Cancer Care Ontario; Coordination and navigation; [cited 2017 November 29];[about 4 screens]. Available from: https://www.cancercareontario.ca/en/cancer-care-ontario/programs/screening-programs/ontario-breast-obsp

[31] Cunningham CT, Cai P, Topps D, Svenson L, Jetté N, Quan H. Mining rich health data from Canadian physician claims: features and face validity. BMC Research Notes 2014;7:682 10.1186/1756-0500-7-682 PMC4193126 25270407

[32] Asch SM, Sloss EM, Hogan C, Brook RH, Kravitz RL. Measuring underuse of necessary care among elderly Medicare beneficiaries using inpatient and outpatient claims. JAMA 2000;284:2325-2333. 10.1001/jama.284.18.2325 11066182

[33] North American Association of Central Cancer Registries (NAACCRS). [Internet]. Certified Registries. [cited 2018 June 10];[about 1 screen]. Available from: https://www.naaccr.org/certified-registries/

[34] World Health Organization (WHO). [Internet]. Cancer incidence in five continents Volume X: indices of data quality. [cited 2018 June 10];[pdf file]. Available from: http://ci5.iarc.fr/CI5I-X/old/vol10/I_09.pdf

[35] Canadian Institute for Health Information (CIHI). [Internet]. Submit data and view data standards. [cited 2018 June 10];[about 1 screen]. Available from: https://www.cihi.ca/en/submit-data-and-view-standards

[36] Sergerie F. Demography Division Statistics Canada. [Internet].Report on the Demographic Situation in Canada Internal migration in Canada from 2012/2013 to 2014/2015. [cited 2018 June 10];[about 21 screens]. Available from: https://www150.statcan.gc.ca/n1/pub/91-209-x/2016001/article/14650-eng.htm

[37] Wilkins R. PCCF+ Version 5K Users Guide (Geocodes/PCCF).Automated Geographic Coding Based on the Statistics Canada Postal Code Conversion Files, Including Postal Codes to May 2011 (Catalogue 82F0086-XDB). Statistics Canada: Health Analysis and Measurement Group, Ottawa, Ontario, Canada, 2012.

[38] Collaborative Stage Data Collection System [Internet]. Chicago: American Joint Committee on Cancer; About CS; [cited 2017 November 29];[about 1 screen]. Available from: https://cancerstaging.org/cstage/about/Pages/default.aspx

[39] Statistics Canada. Canadian demographics at a glance. 2nd ed. Catalogue no. 91-003-X. [Report on the Internet]. Ottawa: Demography Division, Statistics Canada; 2016 [cited 2017 November 29]. 81p. Available from: http://www.statcan.gc.ca/pub/91-003-x/91-003-x2014001-eng.pdf?contentType=application%2Fpdf

[40] The Johns Hopkins ACG® System, Version 10.0. Johns Hopkins University School of Hygiene and Public Health: Johns Hopkins University, 2011.

[41] Charlson ME, Pompei P, Ales KL, MacKenzie C. A new method of classifying prognostic comorbidity in longitudinal studies: development and validation. J Chron Dis 1987;40:373-383. 10.1016/0021-9681(87)90171-8 3558716

[42] Deyo RA, Cherkin DC, Ciol MA. Adapting a clinical comorbidity index for use with ICD-9-CM administrative databases. J Clin Epidemiol 1992 45:613-619. 10.1016/0895-4356(92)90133-8 1607900

[43] Lipscomb J, Yabroff KR, Hornbrook MC, Gigli A, Francisci S, Krahn M, et al Comparing cancer care, outcomes, and costs across health systems: charting the course. J Natl Cancer Inst Monographs 2013;46:124-130. 10.1093/jncimonographs/lgt011 PMC3859291 23962516

[44] Barbera L, Seow H, Sutradhar R, Chu A, Burge F, Fassbender K, et al Quality of end-of-life cancer care in Canada: a retrospective four-province study using administrative health care data. Current Oncology 2015;22(5):341-355. 10.3747/co.22.2636 26628867 PMC4608400

[45] Gigli A, Warren JL, Yabroff KR, Francisci S, Stedman M, Guzzinati S, et al Initial treatment for newly diagnosed elderly colorectal cancer patients: patterns of care in Italy and the United States. J Natl Cancer Inst Monogr 2013;46(1):88-98. 10.1093/jncimonographs/lgt006 PMC3888186 23962512

[46] Warren JL, Barbera L, Bremner KE, Yabroff KR, Hoch JS, Barrett MJ, et al End-of-life care for lung cancer patients in the United States and Ontario. J Natl Cancer Inst 2011;103(11):853-862. 10.1093/jnci/djr145 21593012 PMC3115676

[47] Sibbald B. Rx for data-rich, access-poor researchers. CMAJ [Internet]. 2015 [cited 2015 June 22]; [about 1 screen]. doi:10.1503/cmaj.109-5090 Available from: https://www.prhdn.ca/Other%20Documents/CMAJ%20Rx%20for%20data-rich,%20access-poor%20researchers.pdf 10.1503/cmaj.109-5090 PMC4527900 26100846

[48] National Cancer Institute. [Internet]. Seer-Medicare Linked Database. [cited 2018 June 10];[about 3 screens]. Available at: https://healthcaredelivery.cancer.gov/seermedicare/

[49] Ambs A, Warren JL, Bellizzi KM, Topor M, Haffer SC, Clauser SB. Overview of the SEER-Medicare Health Outcomes Survey linked dataset. Health Care Financ Rev 2008;29(4):5-21. 18773611 PMC4195034

[50] Canadian Network for Observational Drug Effect Studies (CNODES). [Internet]. Data sources. [cited 2018 June 10];[about 2 screens] Available from: https://www.cnodes.ca/about/data-sources/

[51] Kephart G. Canadian Population Health Initiative. Canadian Institute for Health Information. [Internet]. Barriers to accessing and analyzing health information in Canada. [cited 2018 June 10];[pdf] Available from: https://secure.cihi.ca/free_products/CPHI_Barriers_e.pdf

[52] BC Cancer Agency (2011): BC Cancer Agency Registry Data (2011). V2. Population Data BC. Data Extract. BC Cancer Agency(2011). http://www.popdata.bc.ca/data.

[53] BC Vital Statistics Agency (2011): Vital Statistics Deaths. V2. Population Data BC. BC Vital Statistics Agency(2011). http://www.popdata.bc.ca/data

[54] British Columbia Ministry of Health (2011): Medical Services Plan (MSP) Payment Information File. V2. Population Data BC Data Extract. MOH.(2011). http://www.popdata.bc.ca/data

[55] British Columbia Ministry of Health (2011): Consolidation File (MSP Registration & Premium Billing). V2. Population Data BC Data Extract. MOH.(2011). http://www.popdata.bc.ca/data

[56] Canadian Institute for Health Information (2011): Discharge Abstract Database (Hospital Separations). V2. Population Data BC Data Extract. MOH.(2011). http://www.popdata.bc.ca/data

